# Beware bisection: the direction of time-numerosity interactions depends on objective reference

**DOI:** 10.1007/s00426-026-02383-6

**Published:** 2026-09-16

**Authors:** Tutku Öztel, Candice T. Stanfield-Wiswell, Martin Wiener

**Affiliations:** Department of Psychology, George Mason University, 4400 University Drive, Fairfax, VA 22030 USA

**Keywords:** Time perception, Magnitude representations, General magnitude system, Pacemaker accumulator model

## Abstract

In time perception studies, one of the most commonly employed tasks across humans and animals is temporal bisection. In this task, subjects must classify intervals presented into “short” and “long” duration categories. Crucially, this classification can be done either in reference to explicitly learned “anchors”, or in a “reference-free” mode in which no anchors are provided and the subject must use an implicit standard – the so-called “partition variant”. Previous work has demonstrated identical performance and modeling between the anchored and partition variants leading to the conclusion that both variants use a common mechanism. Here we report on a series of findings that critically challenge this assumption in which we, quite accidentally, found that the partition variant produces opposite results on a common task – the time-numerosity interaction, in which more numerous dot arrays are associated with longer perceived durations. Instead, we find that the partition variant induces the opposite pattern, where larger numerosities are associated with a shorter reported duration. We suggest this change in direction is the result of the comparison being made, as we tested other tasks (discrimination, reproduction, anchor variant) and found the oft-reported finding of larger numerosities inducing longer reported durations. We further provide a model in which numerosity induces a lengthening of the criterion itself, which can explain the bidirectionality of the effect. We conclude that the partition variant of temporal bisection, while similar to the anchor variant, may provide substantially different results and caution researchers on drawing conclusions based on this task alone and without additional tasks for verification.

## Introduction

The ability to keep track of time is a critical function necessary for survival. Indeed, organisms are capable of regulating and tracking time across a wide range, from milliseconds to hours (Buhusi & Meck, 2005). In humans and animals, a variety of tasks and methods are available to measure how well an individual can measure intervals of time (Wearden, 2016; Vatakis et al., 2018). These tasks have been widely employed for decades, across behavioral, cognitive, and neuroscience studies, in attempts to better understand and model timing functions. Yet, substantial differences between these tasks exist, which can reflect task-specific effects, where an effect observed on one task may not exist for another task. This has led to a common strategy where multiple tasks can be employed, either across sessions or within a given session, in order to ascertain enhanced precision in the observed effect (Zhang et al., 2019). Yet, one caveat to consider in those tasks that seem similar in their design but findings may in fact be revealing very different underlying mechanisms.

Among the tasks used in time perception studies in humans, the most commonly used are ones in which either a comparison is made or an interval is estimated in various ways (Coull & Nobre, 2008) including the verbal/typed estimations, production, and reproduction tasks. For estimation, a stimulus demarcating an interval is presented to the subject (i.e., 5 s) and they must indicate how long they believe it was (i.e. “I thought that was 5 seconds”). For production, subjects are told the interval up front, and then the subject demarcates it themselves, via a motor response. In reproduction, no temporal labels are provided, and instead the subject is shown the interval (i.e., target interval), and they are then asked to reproduce that interval themselves via a motor response. As a result, reproduced durations longer than the target interval would indicate the given interval was perceived to have lasted longer than its actual duration.

Another set of methodological approaches to investigate subjective time perception in humans includes comparison tasks that require subjects to measure intervals of time and provide some judgment about them. Here, the most commonly used variants of these tasks are discrimination, generalization, and bisection. In the discrimination task, the subject is presented with two intervals in succession - a “standard” interval for a set duration on every trial and a “comparison” interval for a different duration, in either order – and then must indicate which one was longer. In the generalization task, subjects are presented with a range of durations and asked if the durations are the same or different than a remembered standard, with that standard being presented at the beginning of the session. In the bisection task, subjects are presented with a range of durations and must categorize them into “short” and “long” groups. In the traditional “anchor variant” of the task, that is, the one that was first developed (Wearden, 1991; Allan & Gibbon, 1991), subjects are initially exposed to the shortest and the longest interval in the set, termed the “anchor durations”, and trained to discriminate between the two. Participants are then instructed to classify the duration that was presented in the current trial as being closer to the short or long anchor duration. In addition to the anchor variant for bisection, a second variant was later developed. Known as the “partition variant”, this version eschews the need for training on anchors by removing them entirely and instead simply presenting intervals to subjects and instructing them to categorize the intervals relative to all previously experienced intervals (Wearden & Ferrara, 1995). As a result, participants tested in the partition variant are asked to classify the presented stimulus durations as being “short” or “long”, rather than being close to experimentally introduced references for what duration should be considered as “short” and “long”. Here, the assumption is that subjects generate an internal reference by generating a running average of the trial history, which stabilizes within a relatively short number of trials (Wearden & Ferrara, 1995; Wiener et al., 2014). In this variant of the bisection task, thus, participants are asked to classify the duration as a short or long duration, based on their personal referents. We note here that the bisection task is also frequently employed in animal studies of time perception, where the assumptions regarding performance and judgments may not match how humans perform the task; yet, as far as we are aware, the partition variant has not been employed in animals (see Kopec & Brody, 2010; Penney & Cheng, 2018, for reviews).

Data obtained from comparison-based tasks including both anchor and partition variants of temporal bisection, as well as the temporal generalization can be analyzed by fitting the proportion of response choices with a psychometric function and deriving the point at which subjects are equally-likely to categorize an interval as short and long, known as the Bisection Point (BP) and the slope of the function normalized by the BP, known as the Weber Ratio (WR, also referred to as the Coefficient of Variation) (Penney & Cheng, 2018). Here, the BP parameter can be used as a proxy for “bias/accuracy” such that the lower values (i.e., leftward shift in the psychometric function) indicate increased probability of “long” response for shorter durations (e.g., Öztel & Balci, 2020). On the other hand, the WR parameter can be used as a proxy for “precision”, such that the lower values indicate higher precision (e.g., Oztel & Balci, 2020). Notably, performance on both the traditional anchor variant and the partition variant are virtually identical (Wearden & Ferrara, 1995, 1996), with the BP and WR exhibiting corresponding values across a range of durations and conditions. This strong correspondence between the two tasks led researchers to suggest that in fact both tasks relied on the same underlying mechanism (Allan & Gerhardt, 2001; Allan, 2002; Kopec & Brody, 2010). That is, in both tasks subjects were calculating an internal reference and using it as a criterion for judgment, rather than holding the anchor durations in memory during the anchor variant. Supporting this was a modeling work showing that such a mechanism could parsimoniously explain performance across the variants (Wearden & Ferrara, 1995). Additional work with electroencephalogram (EEG) and recordings in non-human primates revealed categorization-dependent responses showing that, instead of accumulating information until the end of the interval, ramping-activity in neurons increased only up until the criterion and then would “plateau” until the trial was over (Wiener & Thompson, 2015; Mendoza et al., 2018; Ofir & Landau, 2022).

Based on the above, one could assume that the anchor and partition variants both reflect the same process for time perception, and so the choice of which one to use is either arbitrary, or constrained by other aspects of the task design. If this is the case, experimental designs utilizing these two variants should reveal parallel results such that both variants should reveal similar findings as a function of the same experimental manipulation. Here, we report that these variants can yield very different effects for the same experimental manipulation – not just a positive effect in one and a null in the other as previously documented (e.g., Gil & Droit-Volet, 2011; Zhang et al., 2014; Yates et al., 2012), but in effects that are opposite to one another, which raises the idea that these two variants tap into different cognitive mechanisms associated with subjective time perception.

We report here a series of exploratory experiments that revealed this in time-numerosity interactions utilizing anchor and partition variants of temporal bisection, beginning with the first experiment in which we found the opposite effect to what have been thus far documented in the current literature that we elaborate below. This surprising result then led to a series of follow-up experiments to better understand why it was happening. We present these for the sake of completeness and comparison with previous work, and as a cautionary message for the current literature on subjective time perception that the choice of which variant to use should not be arbitrary, given that these two variants may in fact tap into different cognitive mechanisms underlying time perception.

To begin, our initial experiment was conducted as an investigation of the well-known time-numerosity interaction where larger numerosities were estimated to last longer, regardless of the actual stimulus duration (Hayashi et al., 2013a, Hayashi et al., 2013b; Javadi & Aichelburg, 2012; Dormal et al., 2006; Xuan et al., 2007; Lambrechts et al., 2013; Xue et al., 2025; Chun et al., 2018; Schlichting et al., 2018; Otsuka & Yotsumoto, 2023; Salillas et al., 2019; Sasaki & Yamada, 2017; Alards-Tomalin et al., 2015; Tsouli et al., 2019; Togoli et al., 2021a, b, 2022; Fortunato et al., 2023; Petrizzo et al., 2023). This effect is referred to as “time dilation effect” and operationalized with the leftward shift in the psychometric functions for larger numerosities. Crucially, time dilation falls under the larger category of magnitude-based judgments, in which time, number and space carry continuous/metric characteristics and are thought to be critical for action and motor movement (Walsh, 2003). One account that aims to explain the relationship between the magnitudes in terms of how they are represented in the human brain is A Theory of Magnitude (ATOM; Walsh, 2003), which argues for a partially shared magnitude system to represent metric information. The main prediction of ATOM is a perceptual “more A more B” pattern such that the perceived magnitude in one dimension should be a linear function of the perceived magnitude in the other dimension (Walsh, 2003; Bueti & Walsh, 2009). The time-numerosity interaction is thus one example of this, in which a time interval presented by larger numbers of items on the screen (e.g., a visual dot array) are judged as longer in duration than those with smaller numerosities. Thus, our first experiment was designed to replicate this well-known time dilation finding before launching into a series of studies to investigate this phenomenon; our choice of the partition variant in this case was entirely incidental. As the resulting experiments we will describe took us in a very different direction than originally planned, we omit the original experimental hypothesis. That being said, the classical time dilation hypothesis predicts a leftward shift in the psychometric functions with increasing numerosity, regardless of bisection task variant (i.e., partition or anchor).

## Experiment 1

Experiment 1 was partitioned in two sub experiments including Experiment 1a and Experiment 1b to test whether the effect of time-numerosity interaction depends on where participants mainly look at while estimating stimulus durations. Experiment 1a and 1b followed the same experimental procedure and materials, except that participants in Experiment 1a were explicitly instructed that they could move their eyes freely while looking at the visual stimulus. In Experiment 1b, participants were instructed to keep their eyes on a central fixation cross (see Procedure for details). Both experiments utilized a partition variant of temporal bisection task where short and long reference durations to classify durations as being closer to were not experimentally introduced.

Surveying the literature on time-numerosity interactions, we could not find any studies that explicitly reported whether subjects were required to maintain steady fixation, yet many studies presented a central fixation point. Crucially, at a phenomenological level, the automatic extraction of numerical information from visual scenes, mediated by the approximate number system (ANS, e.g., Buijsman, 2021), provides a compelling explanation for how numerosity can bias our perception of time. The ANS brings about the ability of approximating quantities exceeding the subitizing capacity (roughly 1 to 4 items), where explicit counting is no longer the primary mechanism, which is mainly considered as depending on parallel and automatic information processing (e.g., Trick & Pylyshyn, 1994). Cheyette and Piantadosi (2019) challenged the notion that ANS relies on parallel and automatic processing, by demonstrating overestimation of numerosity as a function of the number of visual items being serially foveated (i.e., “eyes-free”), which did not depend on looking time. Thus, we reasoned that the direction of time-numerosity interaction (i.e., compression or dilation) could be mediated by saccadic eye movements. If this is the case, a leftward shift in the psychometric functions that indicate a time dilation effect would be more pronounced for the eyes-free compared to eyes-fixed condition.

### Method

#### Participants

Forty right-handed college students (*n* = 20 for both Experiment 1a and Experiment 1b, *M*_age_ = 19.9) participated in Experiment 1 in return for course credit. We did not employ any power analysis to determine sample size. Instead, the sample size was determined based on similar studies using temporal bisection tasks (e.g., Levy, Namboodiri & Shuler, 2015). All participants were right-handed, had normal or corrected-to-normal vision and did not have any history of psychiatric or neurological disorder. None of the methods reported here was preregistered. Experiment 1a excluded 1 participant and Experiment 1b excluded 4 participants in total (see Data Filtering).

#### Stimuli and apparatus

The stimuli were composed of seven log-spaced durations (750, 900, 1081, 1299, 1560, 1873, 2250 milliseconds) presented with seven log-spaced dot numerosity (10, 14, 21, 30, 43, 62, 90 dots) arrays. The dot array that represented the numerosities consisted of randomly generated white dots with equal radius (10 pixels) and were presented on a uniform gray background. The dots were randomly spread across the 85% of the visual display in a way that they did not overlap. All stimulus presentation and response recordings were controlled with PsychoPy software. Keypress responses were made via a mechanical keyboard with a 1000 Hz polling rate (CORSAIR K70 MX Red) and stimuli were presented on a 24-inch LED computer monitor (Dell model S2417DG, 2560 × 1440 pixel resolution, 100 Hz refresh rate).

#### Procedure

Experiment 1 employed a temporal bisection task. On each trial, participants were presented with one of the seven numerosities represented with the white dot array stimuli for one of the seven durations where the numerosity and duration pairings were randomized across trials. All numerosity-duration pairings were presented with equal probability. The dot array stimulus stayed on the screen for one of the seven log spaced duration. The numerosity and duration of the stimulus were randomly chosen in every trial. After each stimulus presentation, participants were asked to indicate whether stimulus duration was presented for a short or long duration by pressing “S” or “L” keys on the keyboard, respectively. Participants were instructed to use their left index finger for short (“S” key) and right index finger for long (“L” key) responses. While classifying the stimulus durations, participants were asked to ignore the numerosities and make their judgments solely on their duration.

**Fig. 1:**
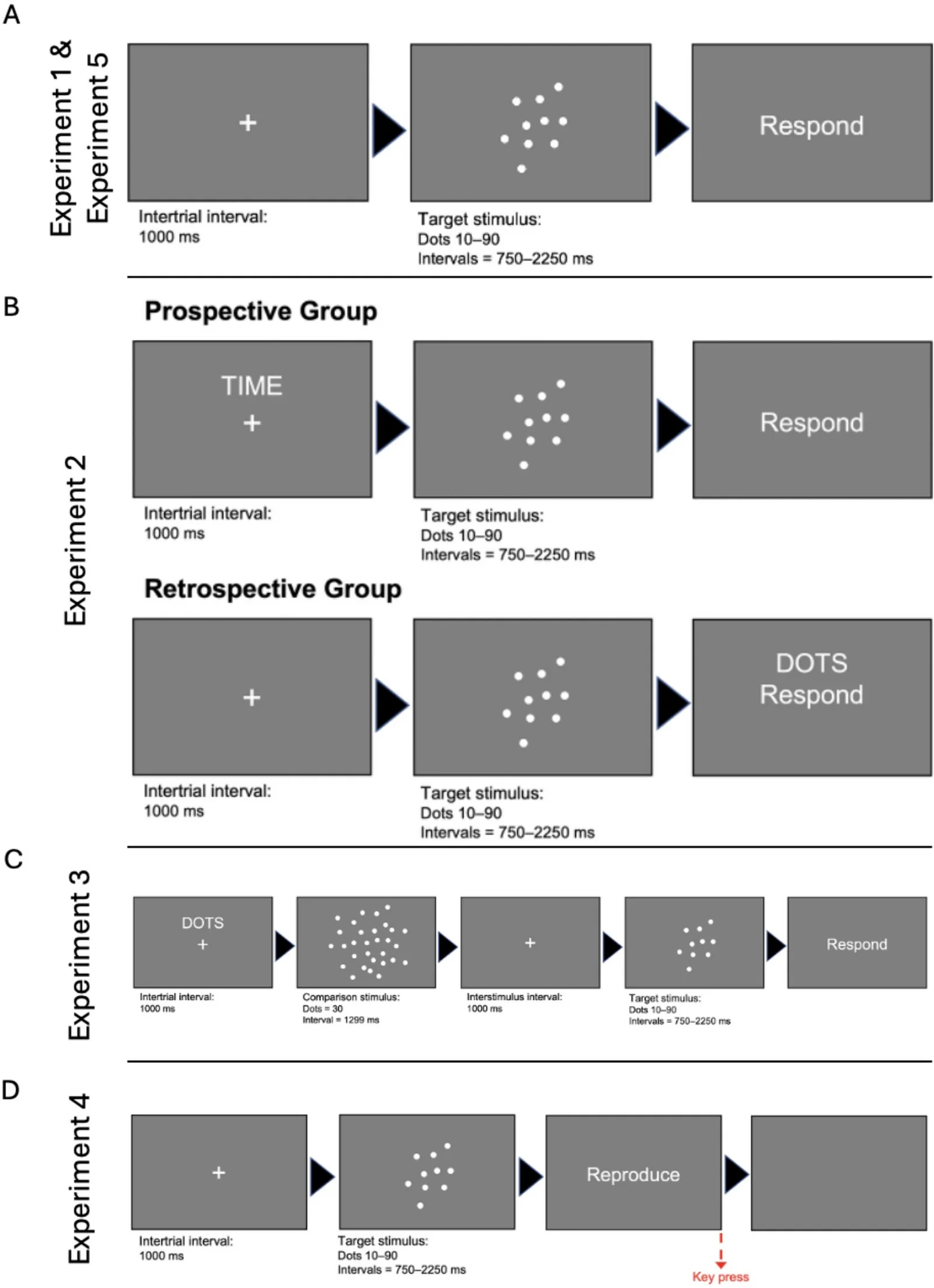
Schematic representation of a trial across different experiments. In all experiments, the time intervals that participants were asked to classify as being closer to “short” or “long” were presented as the duration of the target dot stimulus

Each trial was separated with 1000 milliseconds ITI presented with a central fixation cross. Participants did not receive any feedback regarding the accuracy of their responses and they were instructed not to count or utilize any other strategies that would help estimating the chronometric information (Rattat & Droit-Volet, 2012). The experiment was composed of six test blocks (7 durations x 7 different numbers of dots = 49 trials each block, totaling 294 trials to complete). Participants were allowed to take a break between each block. In Experiment 1a, participants were instructed that they could move their eyes freely during the experiment (i.e., “eyes-free” condition). In Experiment 1b, we explicitly instructed subjects to maintain their eyes on the central fixation while the dot arrays were presented (Experiment 1b, “eyes-fixed” condition), while the rest of the procedural details were the same as in Experiment 1a. We had no eye-tracking available to verify this, yet in post-study interviews all subjects reported they maintained fixation. As a result, Experiment 1 employed a 7 (duration, within subject factor) x7 (numerosity, within subject factor) x2 (eyes fixed/free condition, between subject factor) mixed design. Before the actual experiment, participants completed a practice part for 15 trials. Figure 1A illustrates the flow of an experimental trial.

All participants provided a written consent form before the experiment. All experimental procedures were approved by the local Institutional Review Board (protocol no: 1867674-1). All data and materials associated with the current study will be available upon publication.

### Analytical approach and data filtering

We first calculated the long response probability for each probe duration and each numerosity. We then fitted a two parameter Weibull cumulative distribution function (CDF) to the long response probabilities separately for each numerosity:

$$\mathrm{Weibull}\;CDF\:=\:1-exp(-(x/\lambda)^k)$$

where λ and k were the scale and shape parameters, respectively. We calculated the Bisection Point (BP) as the median of each Weibull fit with the following equation:

$$BP\:=\:\lambda(ln2)^{1/k}$$

Furthermore, we calculated the Weber’s Ratio (WR) as the BP normalized difference limen (i.e., difference limen/BP), where difference limen was calculated as the following equation:

$$\begin{array}{c}\mathrm{Difference}\;\mathrm{limen}=\lbrack\mathrm p("\mathrm{long}")\\=0.75-\mathrm p("\mathrm{long}")=0.25\rbrack/2\end{array}$$

where *p*(long) = 0.75 and *p*(long) = 0.25 depict the duration which was classified as “long” for the 75% and 25% of the times, respectively (calculated as the transformation of the BP as λ(-ln 0.25)^1/k^ and λ(-ln 0.75)^1/k^, respectively). Thus, the BP values captured the bias (or a substitute of intercept) and the WR captured the sensitivity (i.e., slope) of the observer. As a result of these calculations, we obtained BP and WR values for each numerosity (i.e., seven values per parameter in total). Crucially, lower BP values indicate longer perceived durations and thus, time dilation whereas lower WR values depicted higher sensitivity. We used the same calculations for both BP and WR across all experiments.

Low goodness of fit associated with psychometric functions can be indicative of unattentiveness of the participants during the task. For this reason, prior to data analysis, we discarded all fits with R^2^ value below 0.8 (for a similar approach: Oztel et al., 2025). As for the WR, 50% corresponds to at least 50% of change in duration compared to subjective equality points (i.e., BP). This is a very high value for human participants and again, can be indicative of unattentiveness or misunderstanding of response-key mappings or the general task procedure. Taking this into account, we also removed all observations where WR value was larger than 0.5. As a result, ~ 15% and ~ 25% of the total observations were discarded from Experiment 1a and Experiment 1b, respectively.

After this filtering, we examined the shift in psychometric functions as the linear relationship between the numerosities and BP values associated with each numerosity with the following linear mixed effects model:

$$\mathrm{BP}\:\;\:\mathrm{numerosity}+(1\vert\mathrm{participant})$$

where ~ stands for “predicted from” and “(1|participant)” stands for “random intercept across participants”. Accordingly, a decrease in BP values as a function of numerosities would point to a leftward shift, and thus, prolonged perception of stimulus duration. We also used the same linear mixed effects model for WR as a predicted variable.

Given that our aim was to investigate the linear relationship between the predicted variables and numerosity, for the sake of statistical simplicity, we included numerosity (and all other magnitudes, depending on the primary task) as a continuous variable or all models described in this study. The analytical approach and data filtering methods were kept the same for all experiments and planned a priori to investigate time-numerosity interaction. All data associated with this study can be found in Open Science Practices (OSF: https://osf.io/expn5/overviewview_only=ee0fa6c34f5d47bc8288dcab49369b53).

### Results

#### Experiment 1a: eyes-free

**Fig. 2:**
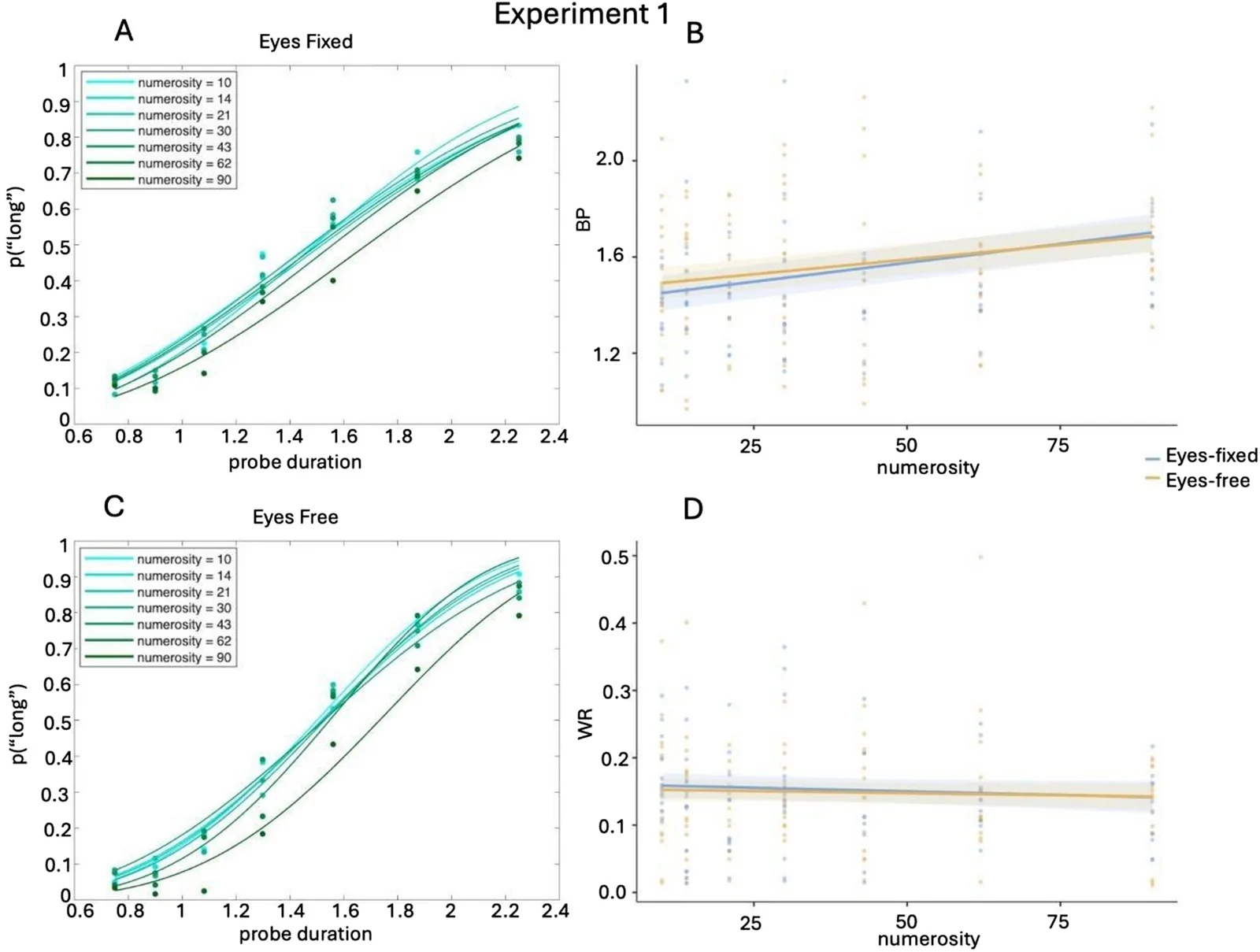
*Left panel*: Average psychometric functions associated with (**A**) “eyes-fixed” and (**C**) “eyes-free” conditions for Experiment 1. *Right panel*: The relationship between numerosity and (**B**) BP and (**D**) WR across experimental conditions for Experiment 1. Shaded areas depict the standard error (SE)

Linear mixed effects model revealed a significant positive main effect of numerosity on BP values (ß = 0.00244, *SE* = 4.43e-4, 95% CI = [0.00156 0.00332], *p* < 0.001), pointing to a rightward shift in psychometric functions as a function of increased numerosity (Fig. 2). This result suggests a shorter perceived time with increased numerosity and thus, opposes the classical time dilation effect as a function of increased numerosity. In contrast, the WR remained constant across the numerosity levels (ß = −1.26e-4, *SE* = 2.44e-4, 95% CI = [−6.09e-4, 3.58e-4], *p* = 0.607).

#### Experiment 1b: eyes-fixed

In Experiment 1b, we observed the same results as we did in Experiment 1a (Fig. 2), a significant positive main effect of numerosity on BP values (ß = 0.00315, *SE* = 4.74e-4, 95% CI = [0.002 0.0041], *p* < 0.001), and no effect on the WR (ß = −2.16e-4, *SE* = 2.93e-4, 95% CI = [−7.99e-4, 3.67e-4], *p* = 0.464). These results, again, point to a shorter perceived time with increased numerosity while pertaining the sensitivity.

### Experiment 1: interim discussion

Experiment 1a demonstrated a rightward shift in the psychometric functions such that the BP values increased as a function of numerosity. This result pointed to an unexpected compression of time perception as a function of increased numerosity, in a “more A less B” perceptual pattern. Additionally, the WR values did not change across the numerosity levels. This differential effect of numerosity on temporal accuracy as indexed by BP and temporal sensitivity is in line with the previous research (e.g., Shukla & Bapi, 2021), highlighting the distinctiveness of the two metrics. For example Shukla and Bapi (2021) tested participants in a temporal discrimination task where they were presented with a standard duration of 550 milliseconds presented with “5” digit and asked to report whether a test stimulus presented with a different Arabic numeral (i.e., 1, 5 and 9) was shorter or longer than the standard duration. Their results demonstrated that while PSE changed across different numeral conditions, WR remained intact, highlighting that these two parameters associated with temporal percepts are not necessarily correlated.

Experiment 1b revealed comparable findings to Experiment 1a, such that the more numerous stimuli perceived to last shorter. Taken together, Experiment 1 suggests that the time-numerosity interaction that yields “more A more B” pattern that was previously documented in the literature (e.g., Javadi & Aichelburg, 2012) does not depend on where participants look at the to-be timed visual stimulus.

The effect observed in Experiment 1 could have been a result of attention being diverted away from temporal processing towards the visual properties of the stimulus. Such an attentional diversion could predict temporal compression (see also attention-gate model, Zakay & Block, 1995). However, attentional diversion away from the stimulus would also have predicted an increase in the WR (Ulrich et al., 2006; Brown, 1997), which was not observed. This explanation also opposed the ATOM/Generalized Magnitude Theory, which argues holistic magnitude information processing, rather than the segregation of stimulus properties that carry different metric information (Walsh, 2003).

## Experiment 2

Given the critical involvement of attention allocation on time perception argued by previous theoretical frameworks (e, g., attentional gate model, Zakay & Block, 1995), we reasoned that the opposing findings obtained in Experiment 1 may have resulted from the numerosity values not being incorporated into the duration estimates enough to induce a time dilation effect. Indeed, recent work by Togoli and colleagues (2022) found that the time numerosity interaction only occurred when numerosity information was “incorporated” into the to-be-timed stimulus; further, when numerosity information was not bound, an opposite effect occurred. We therefore reasoned that to achieve a positive time-numerosity interaction we needed participants to explicitly pay attention to both the numerosity and the duration of the stimulus. This could also require more cognitive resources, which has been documented to dilate time perception (e.g., Im & Varma, 2018). The second experiment thus aimed to examine the effect of attending single (i.e., either time or numerosity) versus dual (i.e., both time and numerosity) features of the stimuli. To this end, we tested participants in a dual task paradigm where they either (1) knew what psychometric feature would be relevant to attend to up front (i.e., prospective condition) or (2) they did not know until the end of the trial so they had to attend to both numerosity and duration features (i.e., retrospective condition). Crucially, we also manipulated whether participants knew which dimension they had to attend to; we reasoned that this situation would ensure subjects jointly attended to both features simultaneously, and so would produce a larger dilation effect.

### Method

#### Participants

A total of forty college students (*n* = 20 per group; 26 female, *M*_age_ = 20.12) participated in Experiment 2 in return for course credit. Following our data filtration procedure (see Data Filtering), we discarded data from two participants (one from prospective and one from retrospective conditions, see Procedure).

#### Stimuli and apparatus

All stimuli and apparatus were the same as described in Experiment 1.

#### Procedure

The general procedure was the same as described in Experiment 1 which employed a bisection task for both time and numerosity. Different from Experiment 1, participants were tested in a dual task paradigm where participants either classified the duration or the numerosity of the stimulus as being short/less or long/more. Participants were randomly assigned to either “prospective” or “retrospective” group (n = 20 each group). Participants in the prospective condition were prompted with the primary task information (i.e., either “TIME” or “NUMEROSITY”) on top of the central fixation cross presented before the stimulus, indicating that they should classify either the duration or numerosity of the stimulus, respectively. On the other hand, participants in the retrospective condition were prompted with the primary task information after the stimulus presentation and at the “Respond” screen (presented on top of the “Respond” text). Each primary task was tested with equal probability (i.e., 7 numerosities x 7 durations x 6 blocks = 294 trials per task, 588 trials in total) and randomized across trials in each group. As a result, Experiment 2 employed a 7 × 7 × 2 × 2 mixed design (7 (duration, within subject factor) x7 (numerosity, within subject) x 2 (task: numerosity or duration, within subject factor) x 2 (prospective, retrospective, between subject factor). Participants received no feedback on accuracy and used the same response mappings as used in Experiment 1 (i.e., “s” button to indicate “short (duration)/few (numerosity)” and “l” button to indicate long (duration)/more (numerosity)”). Participants completed 28 practice trials before proceeding to the actual experiment. Figure 1B illustrates an example trial in Experiment 2.

### Analytical approach and data filtering

We followed the same data filtering approach as we employed in Experiment 1. As a result, ~ 34% and ~ 15% of the total observations were removed from time and number conditions, respectively.

As in Experiment 1, we investigated the shifts in psychometric functions with the following linear mixed effects model, separately for temporal and numerosity bisection tasks:

$$\mathrm{BP}\:\;\:\mathrm{magnitude}\ast\mathrm{condition}\:+\:1\vert\mathrm{participant}$$

Where magnitude (i.e., either stimulus duration or numerosity depending on the primary task type) and condition (i.e., experimental condition, retrospective or prospective) were included as fixed effects and participants were included as random intercept. “*” indicates interaction term. The model included all lower terms. We also ran the same linear mixed effects model using WR as a predicted variable.

### Results

#### Temporal bisection

For temporal bisection judgments, linear mixed effects model again revealed a significant positive relationship between numerosity and BP associated with time, regardless of the experimental condition (ß = 0.0029, *SE* = 4.94e-4, 95% CI = [0.0019 0.0039], *p* < 0.001). This result is in line with Experiment 1 and suggests a shorter perceived duration as a function of numerosity. Overall, the retrospective condition had higher BP values compared to prospective for temporal bisection (*M*_retrospective-prospective_ = 0.28, *SE* = 0.093, 95% CI = [0.092 0.46], *p* = 0.005), pointing to a more overall rightward shift in psychometric function associated with retrospective condition. However, the relationship between BP and numerosity did not change across experimental conditions (ß_Retrospective-prospective_ = 0.001, *SE* = 9.88e-4, 95% CI = [−9.14e-4 0.003], *p* = 0.296). Together, these results suggest that the effect of numerosity on perceived durations did not depend on what stimulus feature (i.e., time or numerosity in prospective and both in retrospective conditions) that attention was allocated.

**Fig. 3:**
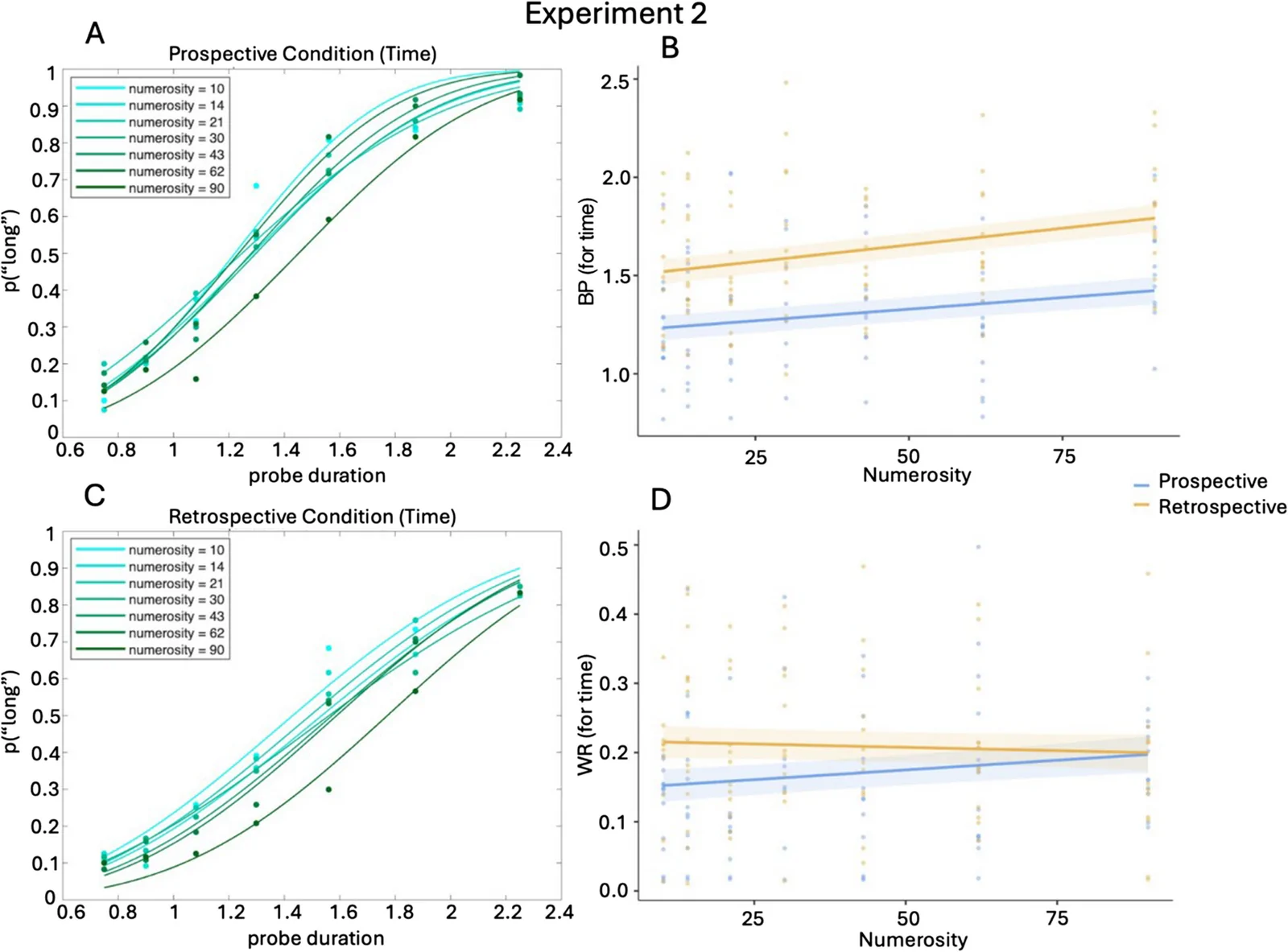
*Left panel*: Average psychometric functions associated with (**A**) “prospective” and (**C**) “retrospective” conditions for temporal bisection in Experiment 2. *Right panel*: The relationship between numerosity and (**B**) BP and (**D**) WR across experimental conditions for temporal bisection in Experiment 2. Shaded areas depict the standard error (SE)

WR associated with the temporal bisection judgments did not change across different numerosities (ß = 1.85e-4, *SE* = 2.16e-4; 95% CI = [−2.43e-4 6.12e-4], *p* = 0.395), which applied to both experimental conditions (ß_prospective - retrospective_ = 7.52e-4, *SE* = 4.33e-4, 95% CI = [−1.02e-4 0.0016], *p* = 0.084). However, WR values were significantly higher for retrospective condition (*M*_prospective - retrospective_ = −0.07, *SE* = 0.035, 95% CI = [−0.139 −0.0018], *p* = 0.048), pointing to a more sensitive temporal bisection performance for prospective condition, regardless of the numerosity levels. The left panel of Fig. 3 shows the average psychometric functions and the right panel of Fig. 3 shows the relationship between numerosity and BP (B) and WR (D) across two experimental conditions.

#### Numerical bisection

For numerical bisection judgments, the BP associated with number did not change across different stimulus durations (ß = 0.432, *SE* = 0.63, 95% CI = [−0.81 1.67], *p* = 0.49); which applied to both experimental conditions (ß_retrospective-prospective_ = −0.143, *SE* = 1.256, 95% CI = [−2.62 2.33], *p* = 0.91). Furthermore, the overall mean BP values across experimental conditions were also comparable (*M*_retrospective-prospective_ = 3.176, *SE* = 3.413, 95% CI = [−3.55 9.9], *p* = 0.36). These results suggest that, as in the case of perceived durations, perceived numerosities did not depend on the stimulus feature that attention was allocated.

**Fig. 4:**
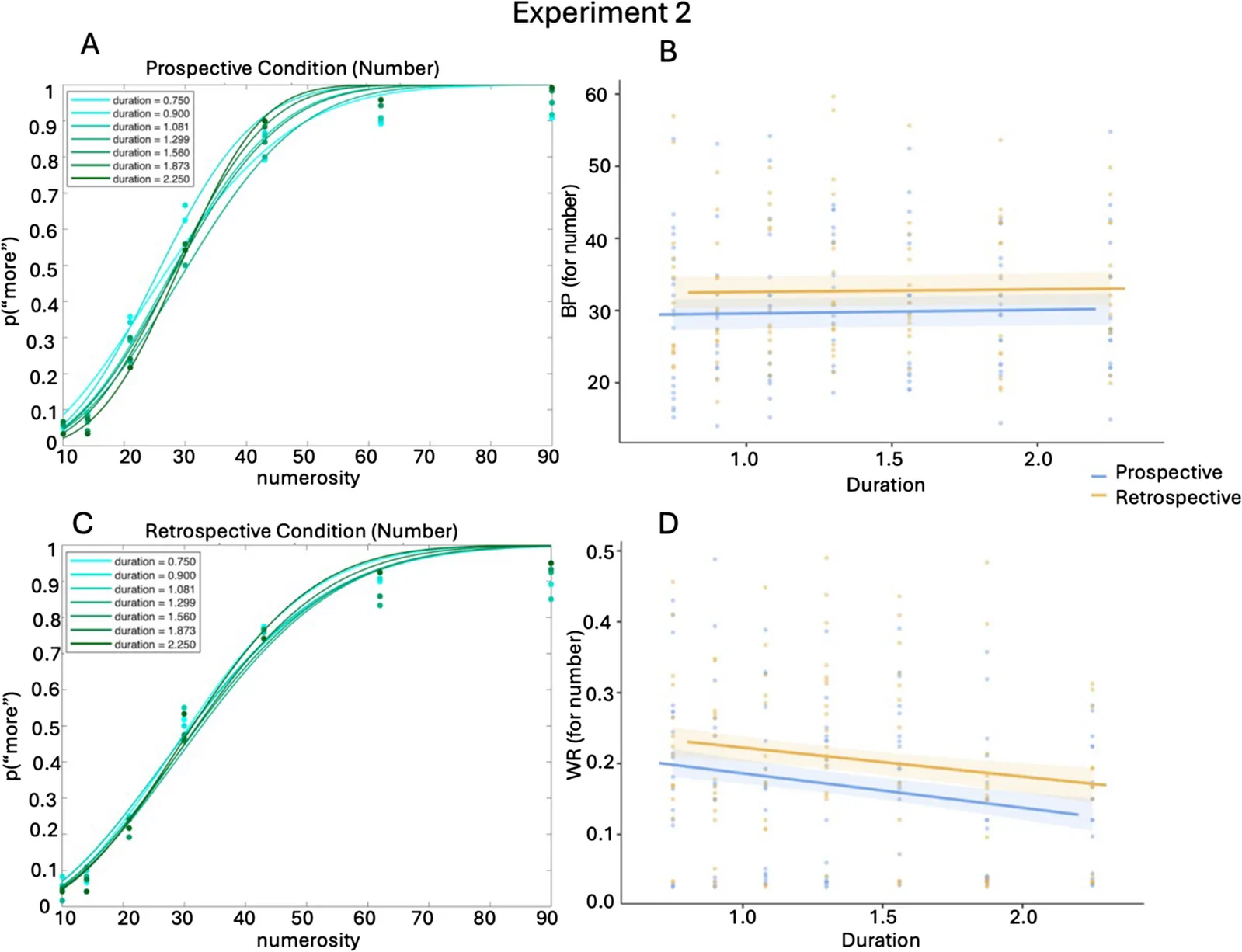
*Left panel*: Average psychometric functions associated with (**A**) “prospective” and (**C**) “retrospective” conditions for numerosity bisection in Experiment 2. *Right panel*: The relationship between numerosity and (**B**) BP and (**D**) WR across experimental conditions for numerosity bisection in Experiment 2. Shaded areas depict the standard error (SE)

WR associated with the numerosity bisection judgments decreased as a function of stimulus durations (ß = −0.045, *SE* = 0.015, 95% CI = [−0.074 −0.016], *p* = 0.003) and this relationship was comparable across experimental conditions (ß_prospective-retrospective_ = −0.0077, *SE* = 0.029, 95% CI = [−0.065 0.050], *p* = 0.79). Finally, the overall WR values were also comparable across experimental conditions, regardless of the stimulus durations (*M*_prospective - retrospective_ = −0.024, *SE* = 0.046, 95% CI = [−0.11 0.066], *p* = 0.60). These results suggest that, as in the case of perceived numerosities, how precisely they were estimated did not depend on the stimulus feature that attention was allocated. The left panel of Fig. 4 shows the average psychometric functions and the right panel of Fig. 4 shows the relationship between duration and BP (B) and WR (D) across two experimental conditions.

### Experiment 2: interim discussion

Experiment 2 revealed that the time-numerosity interaction did not depend on how attention is allocated between numerosity and time. Along with Experiment 1, these results demonstrated that the time-numerosity interaction did not depend on the serial/parallel processing of the numerosity stimulus (Experiment 1) or the differential allocation of attention for the stimulus properties. Additionally, although temporal sensitivity (i.e., WR) was constant across different levels of numerosity, it was significantly higher for retrospective judgments. However, this difference did not apply to numerical sensitivity. Together, these results suggested that, while sharing commonalities, the time and numerosity magnitude domains maintain a certain proportion of unshared variance (Walsh, 2003; see also: Cappelletti et al., 2009, 2011). This differentiation was also observed in the shift in BP, where significant (rightward) shift was observed only for temporal bisection tasks, across numerosity. This asymmetry has been documented by previous studies with different methodological approaches (e.g., stroop task, Dormal et al., 2006; Cappelletti et al., 2009, 2011; Arend et al., 2014). Dormal and colleagues (2006) discusses that the unidirectional effect of numerosity on time perception might have emerged because numerosity information is more automatically processed compared to time perception.

At this point, we reasoned that our findings may not have been the result of how subjects perceived the stimulus, but rather the comparison being made. As noted in the introduction, extant theories of temporal bisection assume that subjects calculate an internal criterion for reference when classifying durations. We therefore reasoned that our findings may have been the result of subjects not making an active comparison to an explicitly presented stimulus.

## Experiment 3

Experiment 3 was conducted using a dual discrimination task, in which subjects sequentially compared a standard duration/numerosity against a range of comparison interval/numerosity values.

### Participants

Forty-two college students (31 females; *M*_age_ = 22.5 years) participated in this study in return for course credit. Five participants were discarded from the total dataset after applying data filtration procedure (see Analytical Approach and Data Filtering).

### Procedure

The data were collected via the Pavlovia online experimentation platform^1^. Pavlovia was chosen for its compatibility with PsychoPy-generated studies, and its superior precision for running studies through an online web browser (Bridges et al., 2020). The experimental procedure was a dual discrimination task where participants compared a target magnitude (i.e., either duration or numerosity, depending on the primary task) with a constant comparison magnitude (i.e., either a reference duration or a reference numerosity, depending on the primary task). As a result, Experiment 3 employed a 7 (durations) x 7 (numerosities) x 2 (task) within subjects factorial design. As in the prospective condition of Experiment 2, in each trial, the primary task was prompted to the participants on top of a fixation cross, which was followed by the comparison stimulus (dots = 30; duration 1299 milliseconds). After the presentation of the comparison stimulus, a central fixation cross was presented for 1000 milliseconds which was followed by the target stimulus differing in numerosity and duration in each trial. All duration-numerosity pairs were randomized across trials and presented with equal probability. Participants were then asked to press “S” or “L” to indicate that the target stimulus was shorter/less or longer/more than comparison, respectively. As for the duration-probability pairs, the primary task was also randomized across trials and presented with equal probability. The experiment took 490 trials to complete (= 7 durations x 7 numerosities x 2 tasks x 5 blocks). Prior to the experiment, participants completed 28 practice trials. Participants used the same response/key mappings as in the previous experiments (i.e., “S” for “short/few”, “L” for “long/more”). Participants did not receive any feedback. Figure 1C illustrates the experimental procedure for Experiment 3.

### Analytical approach and data filtering

Due to highly right skewed WR and BP distributions potentially caused by online experimentation, we performed listwise discard of the observations with WR values larger than 1 and BP values that are between 90% of the lower reference and 110% of higher reference. To keep the discarded data minimum, we did not apply any further filtering based on the R^2^ values. As a result, ~ 19% and ~ 10% of the total observations were discarded for time and numerosity tasks, respectively.

We ran the same linear mixed effects analysis separately for temporal and numerosity bisection and separately for BP:

$$\mathrm{BP}\:\;\:\mathrm{magnitude}\:+\:1\vert\mathrm{participant}$$

where magnitude (i.e., either duration or numerosity depending on the primary task type) is a fixed effect and 1|participant indicates random intercept for participants. We ran the same model separately by including WR as a predicted variable.

### Results

**Fig. 5:**
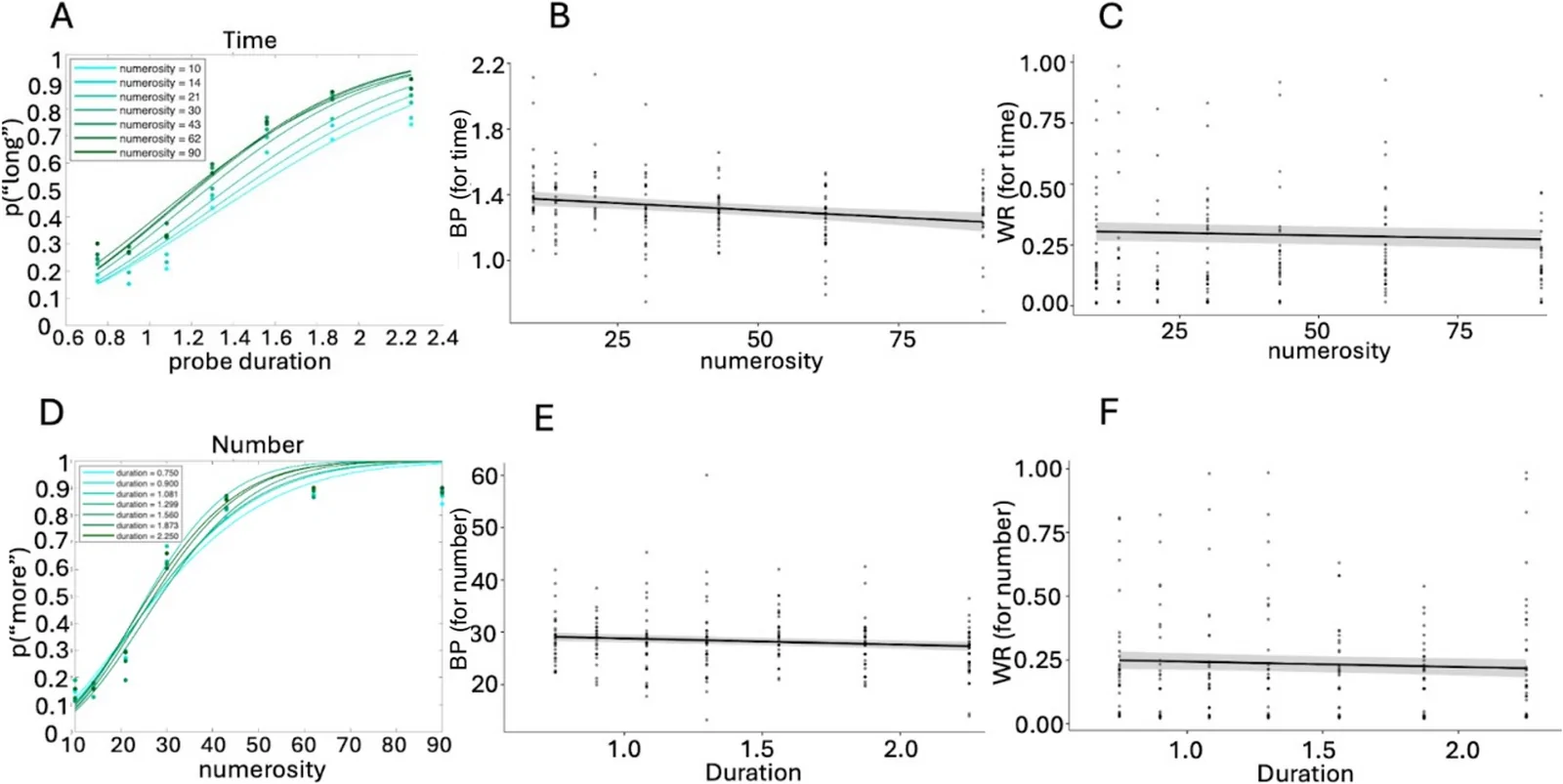
*Left panel*: Average psychometric functions for (**A**) time and (**D)** number. *Middle panel*: The relationship between numerosity and BP for time (**B**) and the relationship between duration and BP for number. *Right panel*: The relationship between numerosity and WR for time (**C**) and the relationship between duration and WR for number. Scatterplots depict datapoints. Shaded areas depict standard error (SE)

Linear mixed effects model revealed a negative relationship between numerosity and BP for time task (ß= −0.0018, *SE* = 4.94e-4, 95% CI = [−0.0027 −7.84e-4], *p* < 0.001). Crucially, this effect applied to numerosity task as well (ß= −1.18, *SE* = 0.59, 95% CI = [−2.35 −0.018], *p* = 0.047), which revealed a leftward shift in psychometric functions for both time and numerosity. However, WR values remained constant across increasing magnitudes for both time and numerosity task (time: ß= −4.06e-4, *SE* = 3.32e-4, 95% CI = [−0.00106 2.49e-4], *p* = 0.223; numerosity: ß= −0.021, *SE* = 0.0198, 95% CI = [−0.06 −0.018], *p* = 0.291). The top row of Fig. 5 illustrates the average psychometric function (A), the relationship between numerosity and BP (B) and WR (C) for time and bottom row of Fig. 5 illustrates the average psychometric function (D), the relationship between duration and BP (B) and WR (C) for number.

### Experiment 3: interim discussion

Experiment 3 demonstrated a *leftward* shift in the psychometric functions, in which the more numerous the visual stimulus is, the longer the duration of the stimulus was perceived. This finding aligns with the “more A more B” perceptual pattern that was predicted by ATOM (e.g., Yamamoto & Miura, 2012; Zhang et al., 2014; see also Zakay & Block, 1995), as well as in line with previous findings in the literature that report a similar effect (e.g., Javadi & Aichelburg, 2012). Notably, this effect also applied for the numerosity judgments such that the longer stimulus duration resulted in higher numerosity estimates. These findings suggested a symmetrical interaction between time and numerosity, which had been reported in previous studies (e.g., Arend et al., 2014; Fabbri et al., 2012; Javadi & Aichelburg, 2012). Taken together, Experiment 3 demonstrated the critical role of memory traces for the representation of the objective reference durations. Furthermore, Experiment 3 reveals differential results compared to Experiment 2 where the objective references associated with target magnitudes were absent. This can be explained by the asymmetrical sensitivity of numerosity and time perception to the objective references.

## Experiment 4

In Experiment 3, we had found the predicted effect, thus replicating previous findings. Yet, since we now had two different tasks, discrimination and bisection, which each provided opposite results, we aimed to address whether the observed opposite direction effects we obtained in the previous experiments occurred due to different comparison demand and thus, differential underlying cognitive processes associated with them (i.e., “close to short or long” in bisection and “same/different” in discrimination). To this end, in Experiment 4 we aimed to eliminate the comparison demand induced via experimental paradigm altogether using a temporal reproduction task. As described in the introduction, reproduction tasks do not rely on semantic labels or decision judgments, and instead require subjects to simply demarcate the interval they were presented.

### Participants

Twenty college students (12 females; *M*_age_ = 21.2 years) participated in this study in return for course credit. Two participants were removed from the formal analysis due to too large CV values (i.e., CV > 0.5, see Analytical Approach and Data Filtering).

### Stimuli and apparatus

The experiment was conducted in a quiet, dimly lit testing room. The stimuli and apparatus were the same as described in Experiment 1.

### Procedure

The task was a temporal reproduction task. In each trial, participants were presented with a dot array ranging from 10 to 90. The dot array stimulus remained on the screen for various durations randomly picked from the seven log spaced duration set, which determined the target duration for that trial. Each duration-numerosity pair was randomized across trials and tested with equal probability. After the presentation of the target stimulus, the word “Reproduce” was prompted at the center of the screen. Participants reproduced the target duration by pressing and holding the space bar for the same amount of the target duration. There was no visual stimulus presented throughout the reproduction. Each trial was separated by an ITI of 1000 milliseconds represented by a central fixation cross. The experiment consisted of 6 blocks and took 294 trials in total to complete (7 durations x 7 numerosities x 6 blocks). Experiment 4 employed a 7 (duration) x 7 (numerosity) within subjects design. Prior to the experiment, participants completed 15 practice trials. Participants received no feedback on their reproduction performance. Figure 1D illustrates a trial in Experiment 4.

### Analytical approach and data filtering

Prior to data analysis, we normalized the reproductions with the target duration (where normalized reproduction = reproduction/target duration) and discarded trials where normalized reproductions were larger than 5. Accordingly, normalized reproductions larger than 1 pointed to overreproduction. No other data filtering was applied. As a result of this filtration, ~ 15% of the observations were discarded.

We ran similar linear mixed effects model as described in Experiment 1 for normalized reproductions:

$$\:\begin{array}{c}\mathrm{Normalizedreproduction}\:\;\:\mathrm{numerosity}\\+\:1\vert\mathrm{participant}\end{array}$$

where normalized reproduction was used as a predicted variable and numerosity was involved in the model as a fixed effect. Intercept was randomized across participants (i.e., introduced as random effect). We ran the same model separately using CV as a predicted variable.

### Results

Linear mixed effects model revealed an increased normalized reproduction as a function of numerosity (ß = 6.42e-4, *SE* = 2.01e-4, 95% CI = [2.48e-4 0.00104], *p* = 0.001), pointing out an overreproduction bias for larger numerosities, irrespective of the target duration.

**Fig. 6:**
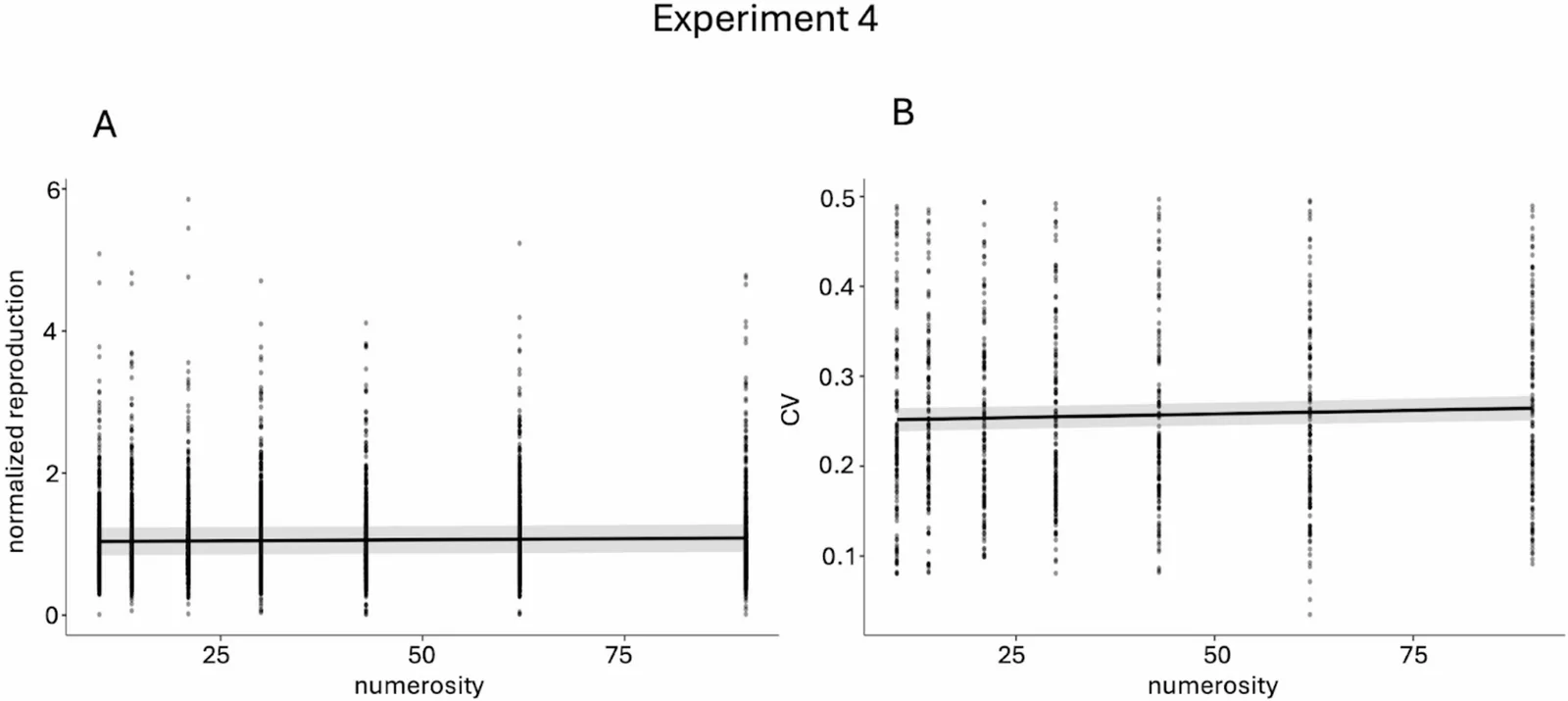
The relationship between numerosity and normalized reproduction (**A**) and CV (**B**) for Experiment 4. Scatterplots depict individual datapoints. Shaded area depict standard error (SE)

We calculated the coefficient of variation (CV) separately for each numerosity and each target duration where CV = *SD*_reproduction_/*M*_reproduction_. Prior to analysis we discarded CV values larger than 0.5. Linear mixed effect model revealed no change in CV as a function of numerosity (ß = 1.60e-4, *SE* = 1.10e-4, 95% CI = [−5.55e-5 3.75e-4], *p* = 0.146). Figure 6 illustrates the relationship between numerosity and normalized reproduction (A) and CV (B).

### Experiment 4: interim discussion

Experiment 4 revealed overreproduction of the reference duration as a function of increased numerosity. As in the case of Experiment 3, this result is in line with the expected “more A more B” perceptual pattern that was predicted by ATOM (Walsh, 2003) and observed previously in the literature. Similar behavioral patterns were observed in previous studies using the reproduction paradigm (e.g., Vicario et al., 2011; Rammsayer & Verner, 2014). Critically, this result reveals that the leftward shift observed in the psychometric function for time in Experiment 3 could not be solely attributed to a potential response bias nor the temporal sensitivity (as reflected in constant CV across different levels of numerosity).

Yet, as a final possibility, we noted that temporal discrimination and reproduction both require subjects to hold an explicitly presented value in memory and compare durations to that value.

## Experiment 5

In Experiment 5, we aimed to test if the observed time-numerosity interaction that yields an expected time dilation effect in “the more numerous, the longer” form depends on the experimentally introduced reference durations that participants need to rely on while classifying durations as being short or long. Therefore, we returned to the bisection task, noting that the anchor variant also provides explicit values for participants to hold in memory and use as a comparison. Thus, in Experiment 5 we replicated the procedure of Experiment 1 except that we provided participants with short and long anchor durations they should base their temporal judgments on.

### Method

#### Participants

Fifty (25 female, 7 did not specify, *M*_age_ = 21.8, 4 did not specify, all right handed) college students participated in this experiment in return for course credit. We decided on sample size as about the double of Experiment 1 to account for factors that are not possible to control for online experiments. After applying data filtration (see Analytical Approach and Data Filtering) we discarded six participants from the dataset.

#### Stimuli and apparatus

All the stimuli were kept the same as in Experiment 1, except that the stimulus presentation and response recordings were done in Pavlovia online experimentation platform (Bridges et al., 2020).

#### Procedure

The procedure was kept identical as in Experiment 1, eyes-free condition, except that participants completed a short training before the experiment to familiarize them with reference durations.

The training included two parts. First, participants were presented with the reference durations of 750 ms (short) and 2250 ms (long) milliseconds. The reference durations were represented with a white square stimulus on the center of the screen to avoid any misrepresentation of the reference durations. After the presentation of each reference duration, participants were randomly presented with each reference duration and were asked to report whether the duration that they observed was the short or long reference duration. Each participant made at least ten such classifications and the training was completed once they reached 80% accuracy for their overall classification performance. Participants received feedback regarding the accuracy of each classification. Upon the completion of the training, participants directly proceeded to the main part of the experiment which was identical with Experiment 1 eyes-free condition and did not receive any feedback. The experiment took about 40 min to complete.

### Analytical approach and data filtering

We excluded all listwise observations where WR was larger than 0.5 and R^2^ value was smaller than 0.8. As a result, ~ 17% of the observations were removed.

### Results

**Fig. 7:**
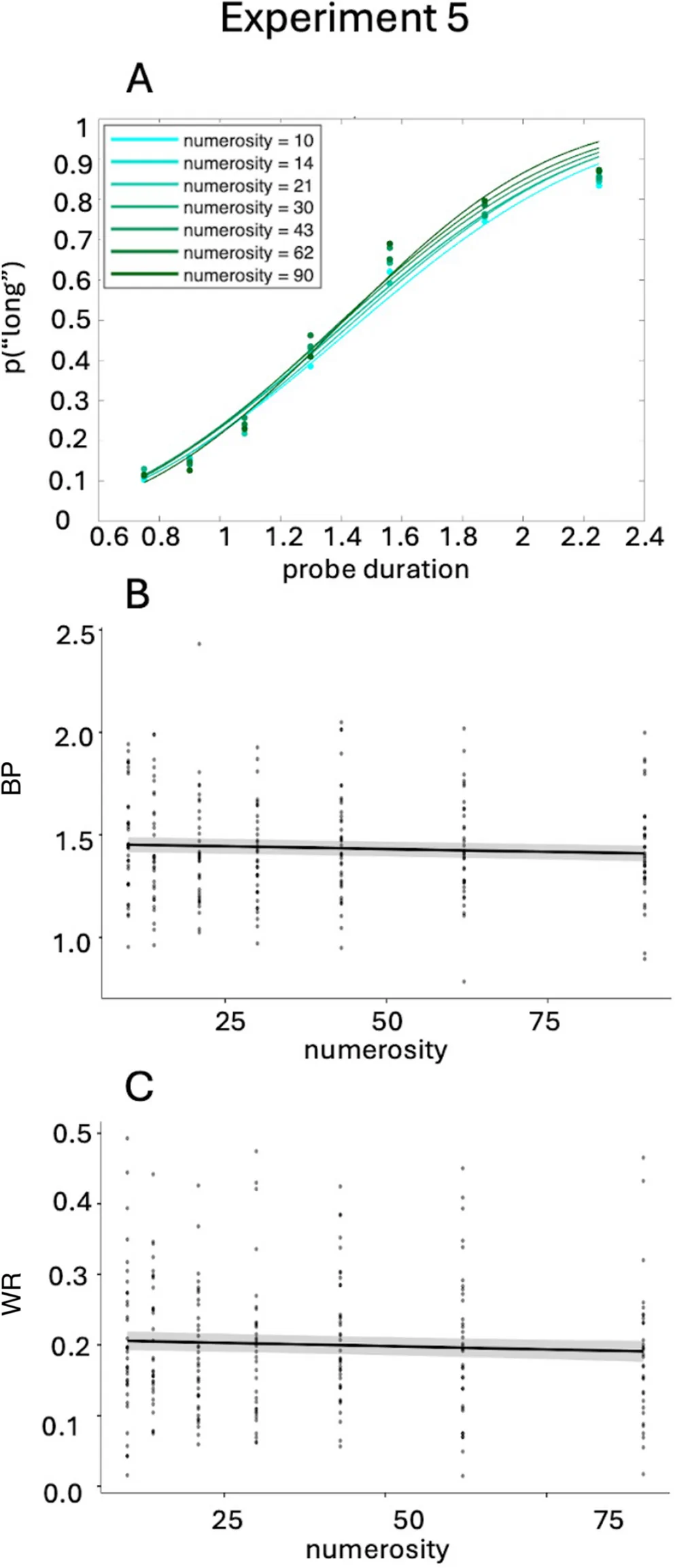
*Left panel*: (**A**) Average psychometric functions in Experiment 5. *Right panel*: The relationship between numerosity and (**B**) BP and (**C**) WR in Experiment 5. Shaded areas depict the standard error (SE)

Linear mixed effect model revealed a significant negative relationship between numerosity and BP (ß = −5.36e-4, *SE* = 2.45e-4, 95% CI = [−0.00102 −5.48e-5], *p* = 0.029), pointing out a time dilation as a function of increased numerosity. However, WR remained virtually constant across different levels of numerosity (ß = − 1.87e-4, *SE* = 1.46e-4, 95% CI = [−4.75e-4 1.00e-4], *p* = 0.201), pointing to a comparable precision across different numerosities. The left panel of Fig. 7 illustrates the average psychometric function (A) and the right panel shows the relationship between numerosity and BP (B, top row) and WR (C, bottom row).

We also directly compared Experiment 1a and Experiment 5 in a single linear mixed effects model. Our results demonstrate that the slope difference that depict the relationship between numerosity and BP for Experiment 1a (i.e., positive relationship) and Experiment 5 (i.e., negative relationship) is statistically significant (ß_Experiment 5 - Experiment 1a_ = −0.003, *SE* = 4.68e-4, 95% CI = [−0.004, −0.0021], *p* < 0.001), underscoring the opposite results obtained by the two experiments.

### Experiment 5: interim discussion

With the final experiment, an overall picture emerged of why the results of the first two experiments were opposite to the predicted effect: that we were specifically using the partition variant of the temporal bisection task. Indeed, when comparing the results of Experiments 1 (partition variant) and 5 (anchor variant), two opposing trends in judgments are observable, with larger numerosity decreasing time estimates in the former and increasing them in the latter. The result is all the more striking when one considers that the trial-by-trial structure is identical; the only difference between tasks is whether participants were exposed to the referents at the outset of the experiment or not.

## General discussion

The current sets of experiments aimed to shed light into how different methodological approaches can affect the direction of time-numerosity interaction. In Experiment 1 and Experiment 2 where participants performed a temporal bisection task without any experimentally introduced reference durations (i.e., partition variant), our results revealed an unpredicted rightward shift in the psychometric functions as a function of numerosity, indicating a time compression effect (i.e., the actual stimulus duration was perceived to last shorter). On the other hand, when reference durations were introduced to the experimental procedure (i.e., anchor variant), this effect was reversed such that the stimulus durations represented with larger numerosities were perceived to have lasted longer, regardless of the task demands (i.e., comparison demand as in temporal discrimination in Experiment 3, temporal bisection in Experiment 5 and no comparison demand as in temporal reproduction in Experiment 4). Thus, together, these results suggest that the time dilation effect as a function of increased stimulus numerosity depends on whether participants were presented with reference durations or were asked to rely on their internal representations of “short” and “long” durations.

Overall, our findings provide a cautionary tale to researchers about drawing firm conclusions of timing effects based on a single task. Indeed, this issue has been raised before, as there are examples in the literature of researchers employing a variety of tasks to study a single phenomenon, finding discrepancies between these results (e.g., Gil & Droit-Volet, 2011; Zhang et al., 2014; Yates et al., 2012; Petrizzo et al., 2023). However, these documented discrepancies involve “positive for one - null for other” type of results across different experimental approaches. In our case, we observed a positive finding on some tasks (i.e. higher numerosity induces time dilation) and a negative finding on others (i.e. higher numerosity induces time compression). One possibility would be to appeal to models of attention, such as the Attentional Gate Model (Zakay & Block, 1995), which is a form of pacemaker-accumulator model. Within the Attentional Gate/pacemaker accumulator framework, the results we obtained in the first two experiments might be due to increased salience of the visual features by the increased numerosity. As a result, attention could have been diverted from the temporal information to the visual information of the stimulus, which ultimately resulted in the “more A less B” effect. However, this interpretation cannot explain the “more A more B” effect for the rest of our experiments with temporal discrimination (Experiment 3), reproduction (Experiment 4) and bisection tasks (Experiment 5). Further, a prediction of this model is that, when attention was diverted away from time, there should be a corresponding increase in the WF (Brown, 1997), which did not occur.

An alternative explanation that could solve the above problem is an impact on the representation of the criterion, rather than the comparison, duration that subjects needed to base their temporal decisions itself. That is, in the absence of an experimentally defined objective reference duration, the criterion duration for categorizing intervals was vulnerable to dilation by numerosity (as in Experiment 1 and Experiment 2). This, in return, would yield distorted representations for criterion duration that the temporal decisions are made based on, such that the criterion duration itself might have been represented as being “longer” compared to its intact representations. As a result, if the criterion duration were dilated, subjects would become more likely to judge stimuli as short with larger numerosities (and thus, a ”more A less B” perceptual pattern). Note that this interpretation does not necessarily imply that the experiments with objective reference durations are exempt from reference dilation effects. Hence, the shifts in the psychometric functions observed in the current study could also be attributed to the potential bias in the reference temporal duration (e.g., Levy, Namboodiri & Shuler, 2015).

The biasing effect of numerosities on reference durations has empirical support. For example, Rammsayet and Verner (2014, 2015) tested participants in a temporal reproduction task to investigate size-time illusion (i.e., larger stimuli perceived to last longer). Here, if size-time illusion occurs due to dilation of the reference duration (i.e., criterion representation), instead of the perceived duration, this illusion should only be present when the size manipulation was employed during the presentation of the reference duration (rather than during reproduction phase). Indeed, they found that the size-time illusion only occurred when the size manipulation associated with the timing stimulus was presented during the presentation of the actual reference duration, rather than during the reproduction phase. This finding led the authors to conclude that size-time interactions occur at the level of encoding into a memory representation, rather than at the perceptual level, as this should have produced an effect at either estimation or reproduction phases of the task. A further extension to the time-number illusion by these same authors (Rammsayer & Verner, 2016), using Arabic digits that varied in size, found that physically larger numbers also induced longer reproduced durations, but that larger numbers did not have an effect unless subjects were specifically instructed to attend to the digit value (see also Karsilar & Balci, 2019). Similarly, Wiener and colleagues (2018) demonstrated that changes in beta oscillations via transcranial alternating current stimulation (tACS) were associated with a change in the memory criterion, instead of being associated with the clock speed changes. At the behavioral level, Chang and colleagues (2011) demonstrated that Arabic numerals with larger values presented during the encoding resulted in prolonged reproduction, but crucially became reversed in direction when larger values were presented during reproduction (Chang et al., 2011). However, another very similar experiment by Cai and Wang (2014) was only able to replicate the encoding effect, with no effect during reproduction. In either case, the results suggest and support the notion that interactions between time and numerosity occur at the level of the memory representation. If so, then a dilating effect on the criterion itself for the partition method, where the subject has not been explicitly presented with a reference, would follow.

**Fig. 8:**
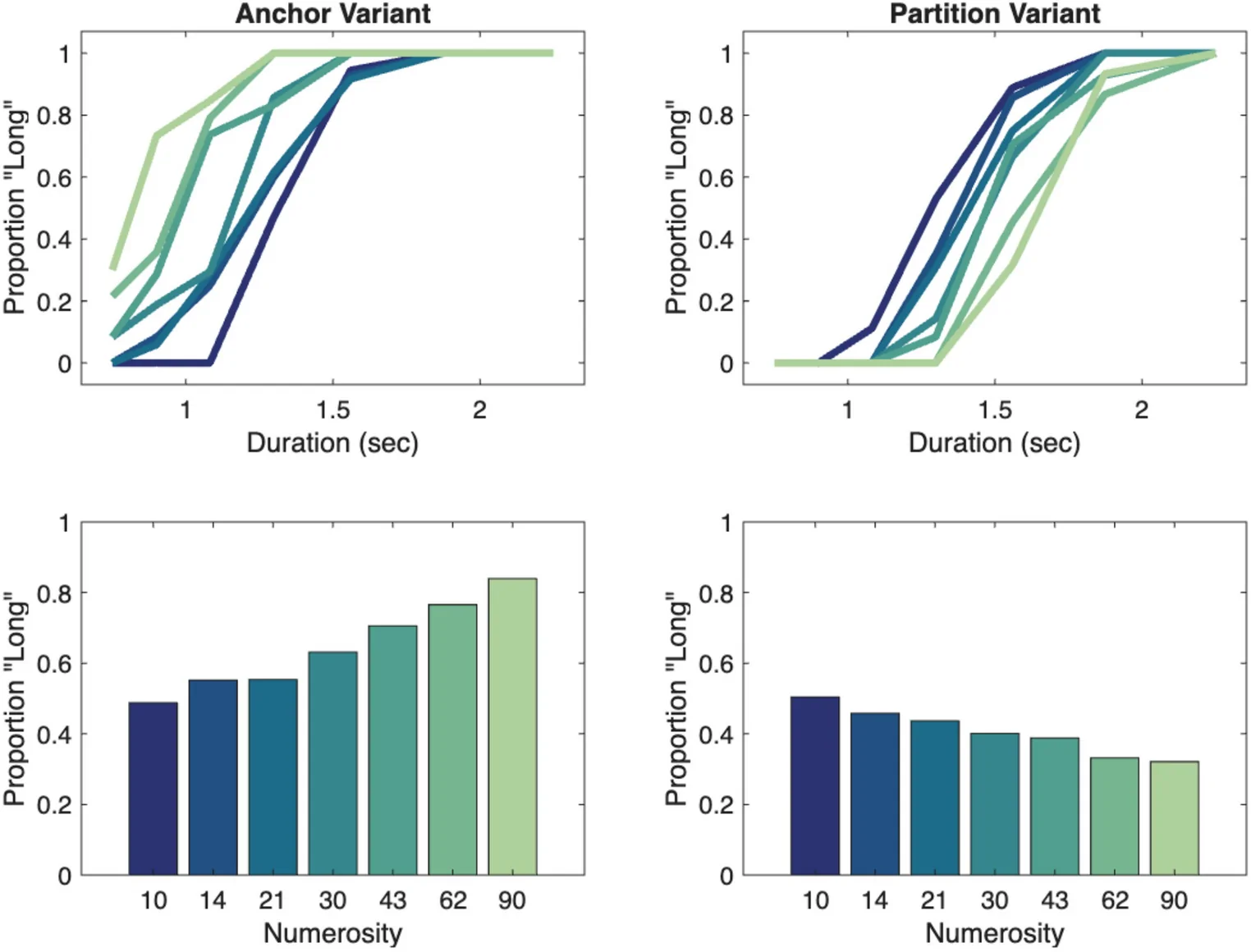
Simulations of a simple scalar timing model in which numerosity differentially affects the criterion (Partition Variant) or the perceived duration (Anchor Variant) on a given trial

We suggest that our findings can thus be explained by a model in which numerosity exerts a differential effect on the criterion and perceived duration, with the partition variant favoring the former and the anchor variant favoring the latter, under the notion that criterion is “immune” in the anchor variant to dilation effects due to an explicitly available reference, leaving numerosity to have a more immediate effect on the comparison interval. To demonstrate, we simulated a simple model of bisection performance, in a manner similar to Wearden and Ferrara (1995) and our previous work (Wiener et al., 2014). Here, perceived duration is set as a draw from a normal distribution with a mean value centered on the true duration and a width set by the WF. Using the geometric mean of the intervals as a simple criterion, we simulated two models: one in which numerosity affected duration directly (anchor variant) and another in which it affected the criterion itself (partition variant). In simulating both models (Fig. 8), a clear dissociation was observed in which numerosity shifts the psychometric functions to the left in the partition variant (dilation effect) and to the right in the partition variant (compression effect).

There are a number of limitations associated with the current study. At the behavioral level, while this study highlights the vulnerability of time-numerosity interaction to different task related parameters, it falls short of elucidating the exact phenomenon underlying the dilated reference duration effects. Further, this study does not directly address why the time-numerosity interaction is actually vulnerable to such task related parameters. Future studies should devise an empirical approach that directly addresses these points. Moreover, whether this vulnerability is generalizable across all magnitude interactions is still unknown. Future studies should test the scope of the obtained effect on different magnitude pairs.

Another important limitation of the current study is that the numerosity stimuli that we used did not explicitly control for other psychophysical dimensions that covary with numerosity, which includes area and density. However, these dimensions positively correlate with increased numerosity. For this reason, this limitation still falls short of explaining the opposite shift in the psychometric functions that we observed in the first three experiments (i.e., rightward shift that indicates shorter perceived duration for more numerous stimuli). On the other hand, this effect still stands as a limitation for Experiment 4 and Experiment 5 where we observe prolonged perceived duration with increased numerosity for the general time dilation effect. However, given that we kept the stimulus set constant for their psychophysical properties across experiments, we still maintain our main standpoint that the methodological differences associated with inclusion of experimental references or not serves as the main reason why we see opposite results among these experiments. Finally, although we asked participants to not count during the experiment, we did not employ any methodological verification to ensure they indeed did not utilize any such chronometric strategies to estimate durations during the experiment. However, if participants counted, one would expect the psychometric functions to retain its central position along the horizontal axis regardless of stimulus numerosity. Thus, the lateral shift (i.e., both right and leftward) we observed in the current study serves as an inherent verification that participants did not utilize counting as a chronometric strategy.

Finally, the use of different testing environments across experiments (e.g., Experiment 1 was tested in the laboratory and Experiment 3 and 5 were tested online) raises the possibility that these experiments are not comparable given the limited controlled factors for online experiments. To address this point, we nearly doubled our sample size in online experiments to correct for uncontrollable factors that you listed. Regardless, while this could indeed raise a compatibility concern regardless of the increased number of participants tested, we have no theoretical explanation as to why the time-numerosity interaction in expected direction (i.e., leftward -instead of rightward- shift in the psychometric functions) would occur in a noisier environment (i.e. online experimentation) instead of a laboratory experiment (e.g., Experiment 1), other than because Experiment 3 and Experiment 5 introduced experimental/objective reference stimuli. For this reason, we think that results obtained from these experiments are fairly comparable regardless of their testing environment (i.e., laboratory or online).

## Conclusion

Overall, our findings suggest that (1) drawing firm conclusions regarding effects on the partition variant of temporal bisection should be cautioned without corresponding evidence from other timing tasks, a rule that should likely be followed regardless of which task is used, just that more than one should be employed and likely ones that use both continuous and discrete responses. (2) The time-numerosity interaction, similar to other magnitude-based interactions with time, likely occurs at the level of representing the criterion duration which is vulnerable to dilation effects, particularly when it is not experimentally provided. We hope that our findings provide a guide for future research and also a cautionary tale when employing the temporal bisection task.

## Data Availability

All data associated with this study can be found in the following link: https://osf.io/expn5/overview? view_only=ee0fa6c34f5d47bc8288dcab49369b53.

## Footnotes

1 These data were collected during the 2020 Covid pandemic, during which lockdown procedures precluded in-person data collection.
